# Furan Dissociation Induced by Collisions with H_3_^+^ and C^+^ Ions

**DOI:** 10.3390/molecules30122559

**Published:** 2025-06-12

**Authors:** Tomasz J. Wasowicz

**Affiliations:** Division of Complex Systems Spectroscopy, Institute of Physics and Applied Computer Science, Faculty of Applied Physics and Mathematics, Gdansk University of Technology, ul. G. Narutowicza 11/12, 80-233 Gdansk, Poland; tomasz.wasowicz1@pg.edu.pl; Tel.: +48-58-347-22-84

**Keywords:** biomass, combustion, irradiation, collisions, furan, ion–molecule reactions, cosmos, radiation damage to cells, ionizing radiation, cations, Balmer decrements, pyridine, tetrahydrofuran

## Abstract

Ion interactions with molecular structures give insights into physicochemical processes in the cosmos, radiation damage, plasma, combustion, and biomass conversion reactions. At the atomic scale, these interactions lead to excitation, ionization, and dissociation of the molecular components of structures found across all these environments. Furan, cyclic aromatic ether (C_4_H_4_O), serves as a gas-phase deoxyribose analog and is crucial for understanding key pathways in renewable biomass conversion, as its derivatives are versatile molecules from lignocellulosic biomass degradation. Therefore, collisions of H_3_^+^ and C^+^ ions with gas-phase furan molecules were investigated in the 50–1000 eV energy range, exploiting collision-induced emission spectroscopy. High-resolution fragmentation spectra measured at 1000 eV for both cations reveal similar structures, with C^+^ collisions resulting in more significant furan fragmentation. Relative cross-sections for product formation were measured for H_3_^+^ + C_4_H_4_O collisions. Possible collisional processes and fragmentation pathways in furan are discussed. These results are compared with those for tetrahydrofuran and pyridine to illustrate how the type and charge of the projectile influence neutral fragmentation in heterocyclic molecules.

## 1. Introduction

Ions and molecules are present in many different environments at the same time, which may cause them to interact with each other. They naturally collide in the interstellar medium, driving chemical transformations of planetary atmospheres or molecular clouds [1,2]. Stellar ionic irradiation can also hinder human space missions to Mars and beyond [3], and, therefore, understanding ion-molecule collisions is essential for assessing the risks from cosmic radiation and solar wind exposure to astronauts, cells, and many other biologically and chemically active substances that can be taken away by the spaceship [3,4,5]. Ion–molecule collisions also play an important role in medicine and engineering. A rising number of investigations focus on the effects of ion beams on DNA/RNA [6,7], its building blocks [8,9], and analogs (e.g., furan [10,11,12,13,14,15], tetrahydrofuran [16,17,18,19], pyridine [20,21,22], pyrimidine [20]), which are crucial for understanding cellular damage and advancing treatments such as hadron therapy for cancer [23]. In engineering, ionic irradiation produces luminescence that helps analyze defects in materials like insulators and semiconductors [24], while focused ion beams are used in etching, deposition, lithography, and imaging [25,26,27]. Understanding these interactions is also becoming increasingly important in the settings of combustion and the conversion of renewable biomass into bioproducts, where specific collisional reactions may play a key role in biofuel production from biomass [28,29,30].

In the aforementioned context, the furan molecule (C_4_H_4_O) is commonly employed as a simplified analog of deoxyribose and ribose [31]. This stems from the fact that deoxyribose adopts a pyranose conformation in the gas phase, while, in DNA, it exists in a furanose form [32]. The extraterrestrial furanose-based ribose and other bioessential sugars were found in primitive meteorites, suggesting that such sugars could have been delivered to early Earth and may have contributed to the formation of functional biopolymers, such as RNA, thus supporting the idea of natural prebiotic chemistry beyond our planet [33]. The furanose ring was also found in the structures of biologically active compounds, such as vitamin B12 [34] and multifunctional biotin-based dyes [35]. Many furan derivatives exhibit promising therapeutic potential. For instance, furan-based compounds such as nitrofurantoin are widely used as antibiotics [36]. Furan rings also play a role in the design of prodrugs that become active under specific physiological conditions, such as the oxidative environments found in tumors [37]. Due to their π-electron systems, furan rings are employed in fluorescent probes and biosensors, where their interaction with specific biomolecules or ions can lead to measurable changes in optical properties [38,39]. Additionally, furan-based monomers, such as furfural and 5-hydroxymethylfurfural (5-HMF), derived from biomass sources like sugars, are utilized in the production of renewable plastics and resins [40], providing sustainable alternatives to petroleum-derived chemicals. Beyond these applications, furan and its derivatives are significant in combustion chemistry, serving as second-generation biofuels derived from the degradation of lignocellulosic cellulose [41,42], and in food and nutrition engineering, where they are formed through the thermal degradation of commercial foods [43,44].

Given the significance of furan molecules across these biological and technological applications, they are excellent candidates for characterizing the mechanisms of ion–molecule reactions. However, a limited number of studies have been conducted on the ion-induced fragmentation processes of the furan ring. Experimentally, these studies have been carried out employing collisional techniques that detect both ionized and neutral products formed during the ionic irradiation of furan. Specifically, Erdmann et al. [11] combined quantum chemical calculations, statistical mechanics, and mass spectrometric collisional techniques for modeling the internal energy redistribution of ionized furan molecules and predicting their dissociation pathways and fragmentation patterns under 100 eV electron and heavy O^3+^, O^6+^, Ar^+^, Ar^11+^, Xe^25+^ keV ions impact. This study demonstrated that the fragmentation pattern of furan is primarily determined by the internal energy distribution it receives rather than the method of excitation or ionization (e.g., ion–molecule collision or electron impact). This supports the fundamental statistical assumption that excess energy is randomly partitioned among internal degrees of freedom, thereby supporting the broad applicability of the M_3_C approach across various excitation techniques. Later, Ascenzi et al. [14] reported a study on the ion–molecule reactions of H_2_O^+^ and OH^+^ ions with a furan molecule. This experiment showed pronounced cross-sections for the production of protonated furan (furanH^+^) and a radical cation (furan•^+^) at low collisional energies (<1.0 eV). Ascenzi et al. [14] also demonstrated that furanH^+^ undergoes a low-energy ring opening, which leads to fragmentation via C–O and C–C bond cleavage. This behavior suggests that protonation significantly increases molecular fragility, particularly in the sugar-like component, which may enhance radiosensitization.

In contrast to mass spectrometric studies involving high-energy collisions of heavy ions with furan, our works [10,12,15] focused on identifying collisional and fragmentation processes through the detection of luminescence resulting from low-energy (≤2 keV) collisions of furan with light atomic and molecular hydrogen and helium cations (H^+^, H_2_^+^, He^+^, He^2+^), which are ubiquitous in cosmic environments and commonly used in hadron therapy. The fragmentation spectra displayed pronounced production of excited hydrogen atoms and CH radicals [10,12,15]. The projectiles such as H^+^, H_2_^+^, and He^2+^ tend to preferentially produce Balmer series lines of hydrogen, whose intensities decrease with increasing principal quantum number *n* more rapidly than predicted by quantum theory. Moreover, low-velocity He^+^ collisions produced resonance-like structures in dissociation yields, indicating a strong velocity dependence on collisional dynamics [12]. Quantum chemical calculations revealed that this effect arises from the formation of transient [He–C_4_H_4_O]^+/2+^ complexes, which enable resonant fragmentation pathways [12]. Collision-induced emission spectroscopy was also employed to investigate furan isomerization induced by 1000 eV H_3_^+^ and C^+^ ion impacts through the search for OH radical emission [13]. The detection of luminescence from OH(A^2^Σ^+^) radicals, which were not originally part of the furan molecule, indicated that hydrogen atoms within the molecule had moved before it dissociated [13]. This supported recent theories about how furan reorganizes bonds and atom positions before fragmentation. Finding these unusual species provided the first experimental proof of these predictions and demonstrated that collision-induced emission spectroscopy can be a valuable method for studying specific ring skeleton changes during ion collisions.

Here, we have investigated gas-phase ion–molecule collisions between furan molecules and the trihydrogen cation, H_3_^+^, the simplest two-electron three-nuclei system in the universe, and heavy carbon ion, C^+^, participating in high-energy reactions in plasma, certain types of combustion, hadrontherapy, or interstellar media. Apart from the role of the OH(A^2^Σ^+^) radical formation and the underlying dissociation mechanisms we investigated at 1000 eV impact energy [13], the gas-phase interaction of such systems has never been explored to the best of our knowledge. The present work focuses on identifying the emissions from all excited products and unraveling the processes occurring in gas-phase furan molecules under the impact of H_3_^+^ and C^+^ cations using collision-induced emission spectroscopy (CIES). By exploring the interaction of the H_3_^+^ ions at different kinetic energies in the 50–1000 eV range, we probe the collision energy dependence of different emitting products and examine the fate of the likely impact mechanisms. We compare these results with ion-induced fragmentation of tetrahydrofuran and pyridine molecules to reveal patterns in how the type and charge state of the projectile influence neutral fragmentation pathways in organic molecules.

## 2. Results

**Figure 1:**
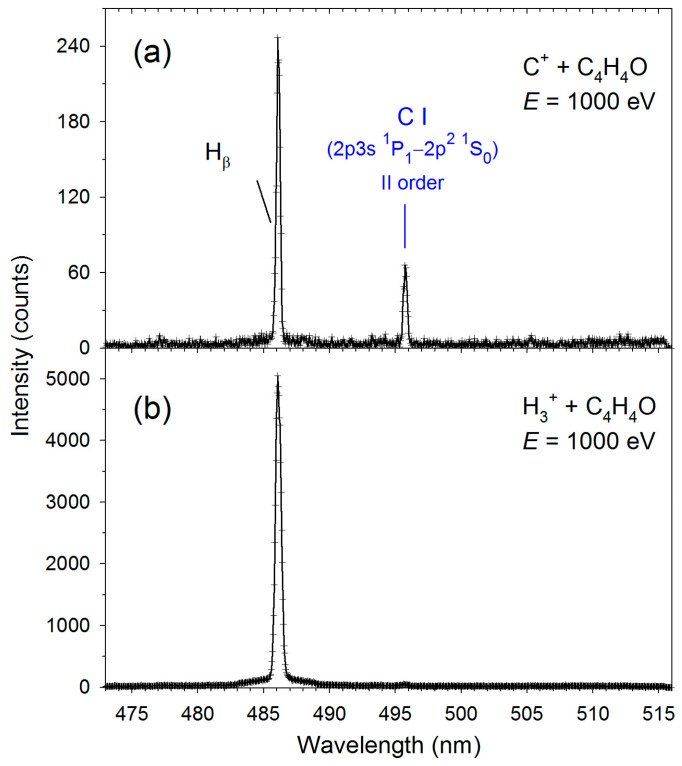
High-resolution fragmentation spectra measured in the 473–516 nm wavelength range for the collisions of (**a**) C^+^+furan and (**b**) H_3_^+^+furan.

**Figure 2:**
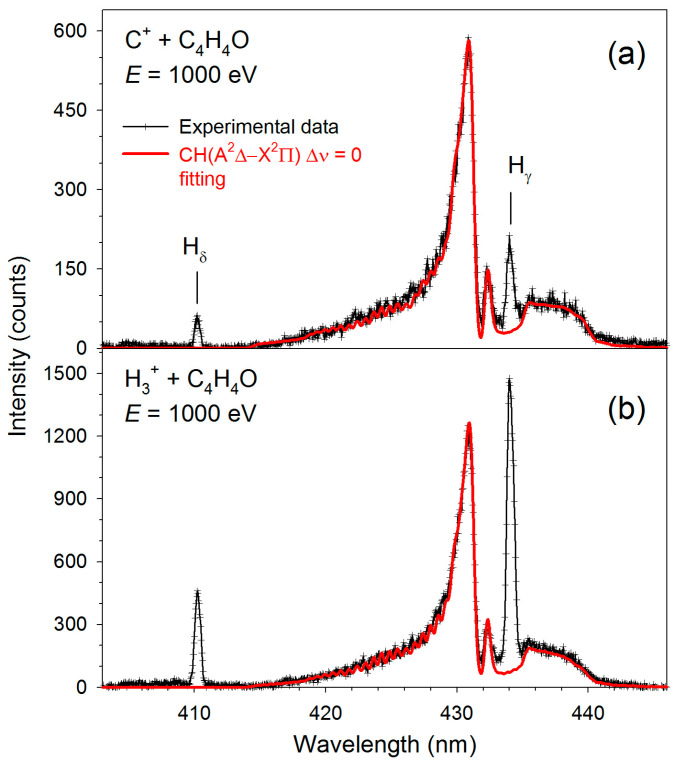
High-resolution fragmentation spectra measured in the 414–446 nm wavelength range for the collisions of (**a**) C^+^+furan and (**b**) H_3_^+^+furan. The solid red lines represent the CH(A^2^Δ→X^2^Π_r_) best fits.

**Figure 3:**
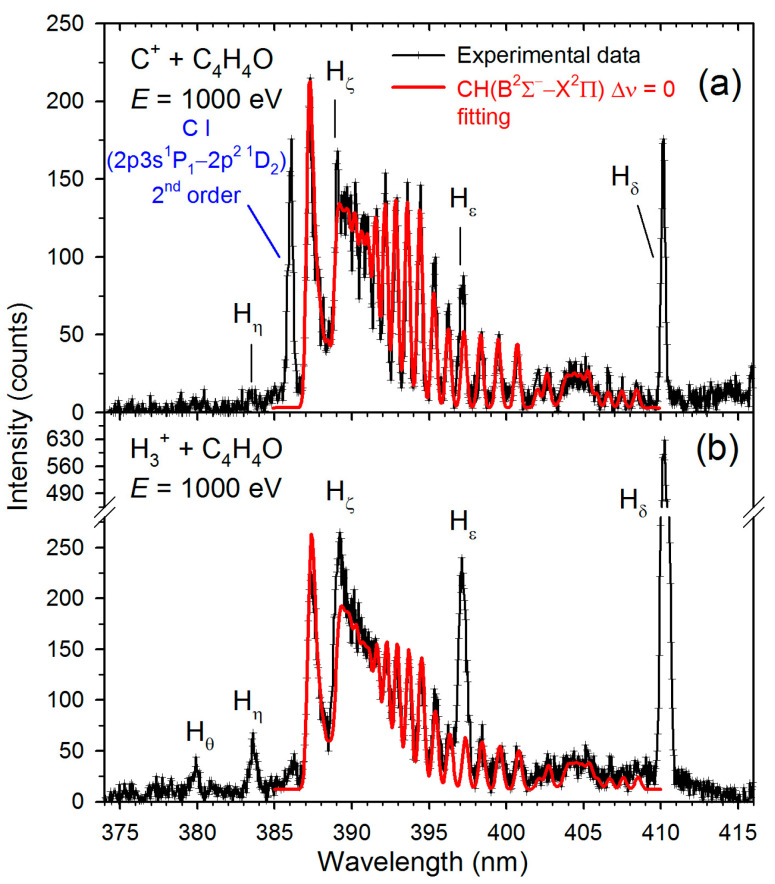
High-resolution fragmentation spectra measured in the 375–415 nm wavelength range for the collisions of (**a**) C^+^+furan and (**b**) H_3_^+^+furan. The solid red lines represent the CH(B^2^Σ^+^→X^2^Π_r_) best fits.

**Figure 4:**
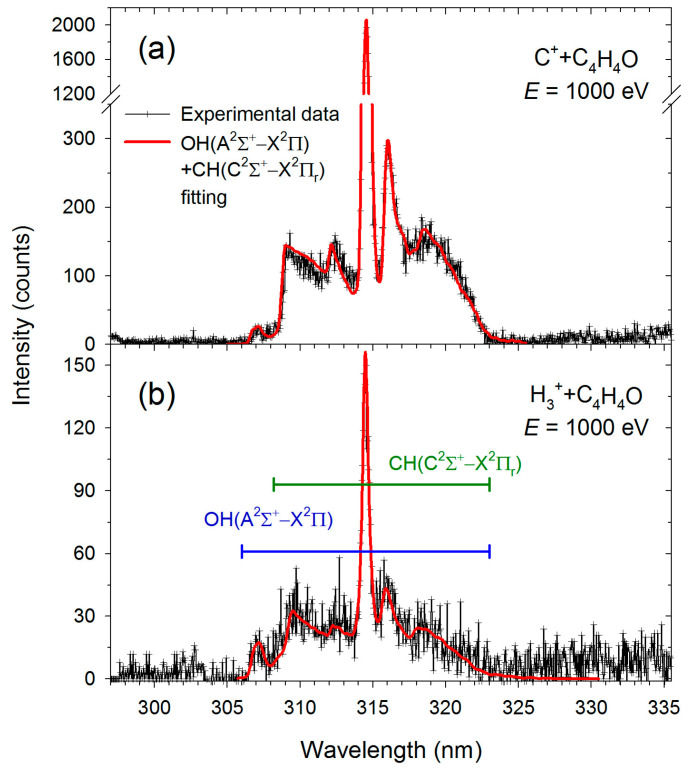
High-resolution fragmentation spectra measured in the 297–335 nm wavelength range for the collisions of furan with (**a**) C^+^ and (**b**) H_3_^+^. The solid red lines represent the OH(A^2^Σ^+^→X^2^Π) + CH(C^2^Σ^+^→X^2^Π_r_) best fits. The experimental and simulated data were taken from [13].

Figure 1, Figure 2, Figure 3 and Figure 4 display the high-resolution optical fragmentation spectra measured at different wavelengths for collisions between furan molecules and the H_3_^+^ and C^+^ cations. For both projectiles, the spectra show structures from the same emitting products. We mainly observe atomic lines of excited hydrogen (Balmer series) and molecular bands of CH radical. However, they differ in relative intensities. Indeed, the Balmer lines have much higher intensities for H_3_^+^ collisions than for C^+^. Specifically, Balmer series lines up to the H_θ_ line (*n* = 10→2) are clearly visible in collisions with H_3_^+^. As the emission of H_α_ (*n* = 3→2) was outside the sensitivity range of our detector, we did not measure its luminescence.

On the other hand, molecular bands dominate the C^+^+furan spectra. However, the shapes of these bands are different than those observed for H_3_^+^ collisions. Therefore, theoretical contours were fitted to the molecular bands using the LIFBASE program [45]. The red solid lines represent these fittings in Figure 2, Figure 3 and Figure 4, and the resultant vibrational (*Tᵥ*) and rotational (*T_R_*) temperatures are listed in Table 1. In addition, spectral lines of neutral carbon atoms appear in collisions with carbon cations (see Figure 1 and Figure 3).

Table 2 shows the positions (λ) of the band heads or the centers of the atomic lines observed in the H_3_^+^ and C^+^ collisions with furan.

**Figure 5:**
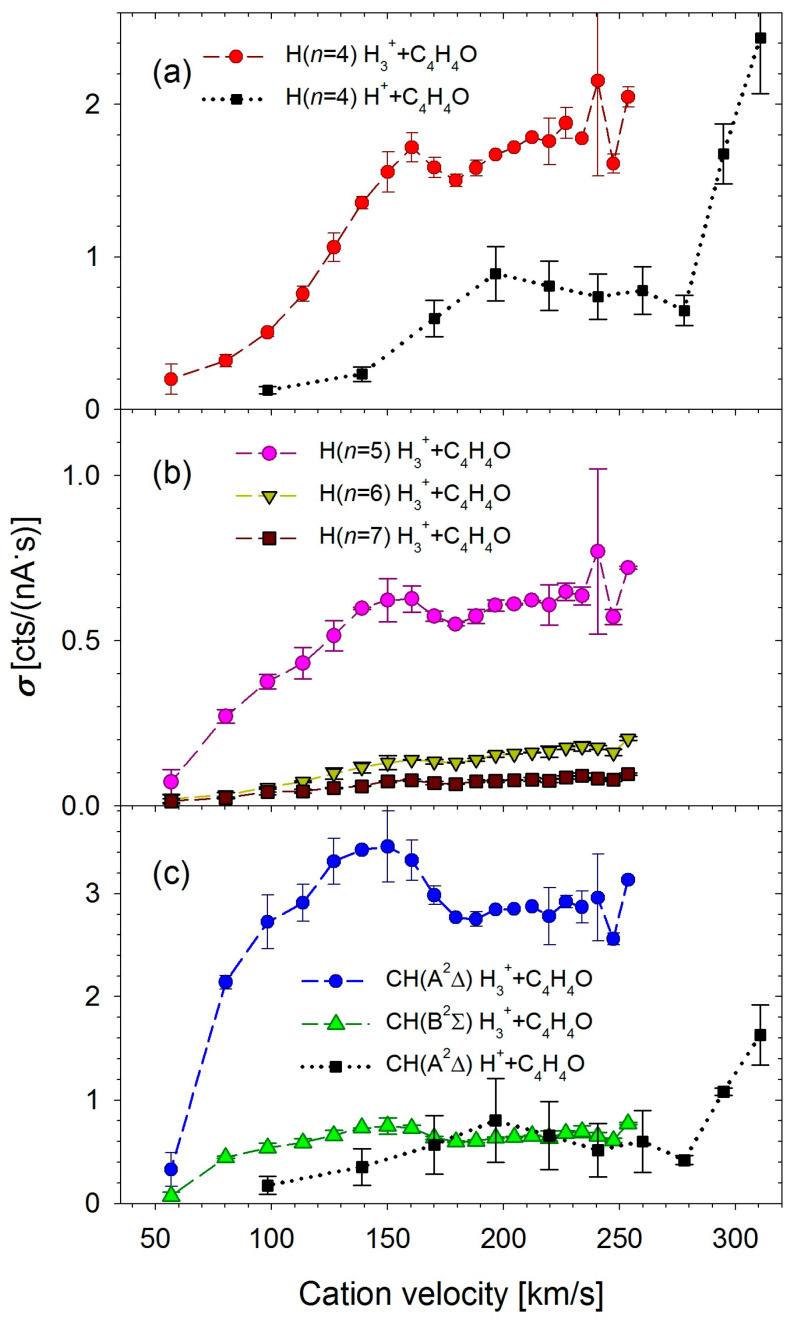
The relative cross-sections for the production of the excited products determined in collisions of H_3_^+^ with furan molecules: (**a**) H(*n*), *n* = 4; (**b**) H(*n*), *n* = 5–7; (**c**) CH(A^2^Δ, B^2^Σ^+^). The experimental data for H^+^ + furan collisions [10] is given for comparison.

Figure 5a–c show the relative cross-sections for the production of the most intensive species identified in collisions with H_3_^+^. These species are detailed in Figure 1, Figure 2 and Figure 3 and listed in Table 2.

The H(*n* = 4) curve gradually increases from the lowest velocities (see Figure 5a). It reaches a small maximum at a velocity of ~160 km/s (corresponding to 400 eV). This curve differs slightly from the H(*n* = 4) yield obtained in furan–proton collisions [10], shown in Figure 5a for comparison. This curve, in turn, reaches its first maximum at a velocity of ~200 km/s (200 eV) and rises swiftly above 280 km/s (400 eV). The cross-sections of the remaining fragments of the hydrogen atom, shown in Figure 5b, reflect the H(*n* = 4) curve.

The cross-sections of CH molecular fragments exhibit a slightly different behavior (see Figure 5c). These curves reach a maximum at a velocity of ~150 km/s for H_3_^+^ ions, corresponding to an energy of 350 eV. Below the maximum, the curves follow a parabolic shape. Above the maximum, the cross-sections decrease until the ions reach a velocity of ~188 km/s (550 eV), after which, at higher velocities, they remain constant within the measurement uncertainty.

The average relative abundances (*RAs*) of the luminescence products were then calculated to identify trends among different impact systems. *RA* is defined as the ratio of the emission yield of each excited fragment to the total yield of all detected luminescent products (180–600 nm range), averaged over the cation energy range (25–1000 eV). Note that *RA* values exclude H_α_ emission, which lies outside the detector’s sensitivity range. Table 3 presents the *RA* values for H_3_^+^ collisions with the furan molecule along with previous *RA* results determined from collisions of different cations with furan [10,12,15], tetrahydrofuran [16,18,19], and pyridine [22] molecules.

## 3. Discussion

Our investigations [10,12,13,15,16,18,19,21,22], and the experimental and theoretical results from other research groups (see, e.g., [6,7,8,9,11,14,17,48,49,50,51]) show that ion collisions with gas-phase molecular targets lead to a series of different collisional processes, resulting in specific fragmentation reactions. These include the following:

(i) Charge transfer (CT);
(ii) Dissociative excitation;
(iii) Dissociative ionization;
(iv) Complexes formation.

Previous studies [8,9,10,12] have shown that the charge transfer process dominates under the cation velocities and projectile types investigated. Furthermore, our experiments reveal only the most prominent signatures associated with the processes in question. A more detailed understanding can only be achieved through quantum chemical calculations, which we suggest as a direction for future work.

The charge transfer reaction (also known as the electron capture mechanism) is one of the primary mechanisms for depositing energy into the target molecule. It happens when an electron is transferred from C_4_H_4_O to the H_3_^+^ or C^+^ cations, leading to the ionization of furan and further fragmentation of its C_4_H_4_O^+^ parent cation. This reaction is generally exoergic [50,51], enabling a considerable amount of energy to be transferred to the internal degrees of freedom of the molecular products. This is also true for H_3_^+^ and C^+^ collisions, where H_3_^+^ + C_4_H_4_O → H_3_ + C_4_H_4_O^+^ releases 4.22 eV, while C^+^ + C_4_H_4_O → C + C_4_H_4_O^+^ delivers 2.38 eV of energy. We used 13.1 [52], 11.26 [53], and 8.88 [54] eV ionization energies of H_3_, C, and furan in these simple evaluations, respectively. Due to its exothermicity, the electron capture mechanism is energetically the most favored channel.

During this reaction, both cations are neutralized. Schlathölter et al. [55] demonstrated that most of the energy released during the electron transfer is deposited onto the projectile. This energy deposition can produce a high yield of neutralized projectiles excited to multiple electronic states. Figure 1 and Figure 3 illustrate the excited carbon atoms most likely generated in the CT reaction. In fact, our previous studies concerning H^+^, C^+^, and O^+^ collisions with tetrahydrofuran [16] showed that the CT mechanism dominated over other processes, leading to the production of large amounts of excited hydrogen atoms in collisions with protons. However, the appearance of intense carbon atomic lines in collisions with carbon ions was unusually amplified. The collisions with heavy cations such as O^+^ did not result in the complete fragmentation of THF. Consequently, carbon atomic lines of negligible intensity were observed. It should be noted that neither electron impact studies on furan [56] nor photodissociation experiments involving furan or other experiments with heterocyclic molecules [57,58,59,60] have demonstrated fragmentation of the target intense enough to yield individual carbon atoms. The fragmentation spectrum observed in C^+^ + furan collisions is more similar to that of O^+^ + THF than to C^+^ + THF. This suggests that the carbon atoms detected in the present experiments may originate from both molecular fragmentation and neutralized C^+^ ions formed via charge transfer processes. In contrast, the highest yield of neutral carbon atom emission in C^+^ + THF collisions is a result of a direct atom knockout process rather than a charge transfer (CT) reaction. Kunert and Schmidt [61] conducted a theoretical study of H^+^, C^+^, and Ar^+^ atomic ion collisions with C₆₀ molecules in the energy range 0.02–0.45 a.u. using nonadiabatic quantum molecular dynamics (NA-QMD). They modeled electronic and vibronic excitations self-consistently and observed knockout-driven fragmentation across various projectile ions and collision energies. This is because, in low-energy collisions, nuclear stopping (Rutherford-like nuclear scattering) dominates over electronic stopping, leading to non-statistical fragmentation in which individual atoms are directly knocked out during the collision [61,62]. These findings are in agreement with those of Chen et al. [63], who employed electronic and nuclear-stopping models to investigate ion–PAH collisions and derived a scaling law for single-carbon knockout cross-sections. At high ion energies (11 keV), electronic stopping dominated, with energy transfer localized in regions of high electron density, such as chemical bonds. At low energies (110 eV), nuclear stopping was the primary mechanism, while electronic stopping contributions were only a few electronvolts.

In H_3_, a single hydrogen atom would need to share its only electron with two other hydrogens, leaving it without enough electrons to complete a full valence shell, which makes H_3_ unstable. Indeed, its lifetime is equal to 300 ps [64]. Therefore, the CT reaction leads to the immediate breakup of the H_3_, usually into H/H_2_ or H/H/H [52,65]. These fragments are usually excited, thus giving rise to the amplified production of hydrogen emissions.

As shown in Table 3, the relative abundances of key fragmentation products, hydrogen (H) and methylidyne (CH), resulting from collisions of furan, tetrahydrofuran (THF), and pyridine with various ion projectiles exhibit distinct trends that reflect both the nature of the projectile and the molecular structure of the target. In furan, the average relative abundances (*RAs*) of hydrogen-emitting fragments are 58.3%, 46.3%, and 37.6% for H^+^, H_2_^+^, and H_3_^+^, respectively. THF exhibits a generally higher hydrogen emission, with corresponding *RAs* of 88.8%, 76.2%, and 67.3%. Pyridine, investigated with H^+^ and H_2_^+^ only, displays a comparable trend to the pattern observed for furan, with hydrogen emission at 61.4% and 45.9%, respectively. In contrast, CH radical emission increases with hydrogen projectile complexity. In furan, CH emission RAs are 39.9%, 51.0%, and 59.4% for H^+^, H_2_^+^, and H_3_^+^. THF shows lower CH emission overall—11.2%, 23.8%, and 32.7%—while pyridine emits CH fragments at 26.9% and 40.4% under H^+^ and H_2_^+^ impact, respectively. Moreover, the He^+^ impact on furan yielded 8.4% hydrogen and 82.1% CH. Pyridine impacted by He^+^ yielded similar hydrogen abundances (10.8%), with the remaining signal attributed to other fragment species, mainly CH (42.2%), CN (27.8%), and C_2_ radicals (13.3%) [22]. For THF, C^+^ and O^+^ collisions produced 4.8% and 17.6% hydrogen emission and 36.2% and 69.6% CH radical emission, respectively. For hydrogen emission, a clear linear decrease in relative abundance is observed with increasing complexity of the hydrogen projectile. The heavier the projectile, the more fragmentation products are observed, suggesting a redistribution of internal energy that favors C–C, C–N, or C–O bond cleavages as the cation size increases, making hydrogen emission less probable. Moreover, THF consistently exhibits higher hydrogen emission and lower CH radical emission compared to furan and pyridine. This is likely due to its saturated structure, higher hydrogen content (C_4_H_8_O vs. C_4_H_4_O in furan and C_5_H_5_N in pyridine), and weaker C–H bonds (3.97 eV [59]) relative to the more rigid, aromatic structures of furan and pyridine, which have C–H bond dissociation energies of 4.46 eV [66] and 4.59 eV [67], respectively.

Fragmentation spectra from He^+^ collisions with furan and pyridine were more structured with more intense emitting products than those from He^2+^ impacts. As indicated, in furan, the He^2+^ impact resulted in 47.9% hydrogen and 52.1% CH emission production, whereas the He^+^ impact yielded 8.4% hydrogen, 82.1% CH, and 8.2% C2 and 1.3% He I emissions [12], indicating a shift in the dominant fragmentation pathways. These observations can be explained based on recent results from heavy ion impacts (O^3+^, O^6+^, Ar^+^, Ar^11+^, Xe^25+^ at keV energies) reported by Erdmann et al. [11]. They showed that as the charge state of the projectile ion increases from O^3+^ to O^6+^ to Ar^11+^, the peak of the internal energy distribution shifts toward lower energies. Higher-charged ions (e.g., Xe^25+^) typically capture electrons at larger impact parameters, meaning they interact at greater distances. Thus, higher-charge-state ions tend to interact with the target molecule at greater distances, resulting in reduced energy transfer. Conversely, lower internal energy transfer corresponds to decreased intensities of product (fragment) ions in the mass spectra, as observed by Erdmann et al. [11]. This implies that the fragmentation yield is directly influenced by the amount of internal energy deposited into the molecule—less energy leads to fewer fragmentation events.

In addition, fragmentation spectra from collisions of helium cations with furan and pyridine displayed more complex structures compared to those from collisions involving hydrogen and heavier C^+^ projectiles. The enhanced fragmentation by He^+^ is attributed to the fact that resonant electron charge transfer is energetically forbidden in He^+^ + molecule collisions. This is due to a significant energy-level offset of 15.7 eV and 16.07 eV between the molecules and the He^+^ ion for furan and pyridine, respectively. Therefore, the He^+^ cations can transfer a large amount of momentum and energy to the target molecules, leading to immediate fragmentation, as predicted for H^+^, C^+^, and Ar^+^ collisions with C₆₀ molecules by Kunert and Schmidt [61]. In contrast, other projectiles enable resonant electron capture, which leads to reduced fragmentation. This is because a large amount of the energy released during the electron transfer process is deposited onto the projectiles [55], yielding a high yield of neutralized cationic species excited to multiple electronic states.

Given that the primary decomposition pathways of furan have been extensively reviewed in the literature [10,11,12,13,14,15,56], we will not discuss them in detail here. However, we would like to briefly discuss a particularly interesting hydrogen migration reaction induced by cation impact.

The skeleton of the furan molecule consists of four CH units and one oxygen heteroatom and contains no OH structural units. The appearance of emission from the excited OH(A^2^Σ^+^) is clear evidence of a chemical bond rearrangement process in which one of the hydrogen atoms moves to O prior to dissociation. It has been suggested [13] that the most favorable pathway for OH formation involves simultaneous 2,1- and 3,2-H shifts along with cleavage of the C(2)–O(1) bond, requiring only 2.66 eV above the canonical furan structure. This pathway leads to the formation of two stable alkyne–alcohol isomers (HCCCHCHOH), which differ by just 0.12 eV and are both capable of releasing OH from the molecular end. An alternative pathway requires higher energy (approximately 1 eV more) and produces a less stable closed-ring furan-C_2_₁-carbene, making it less likely, as it must compete with a barrierless and energetically more favorable C–O bond fission reaction.

The OH(A^2^Σ^+^) luminescence, indicative of the hydrogen atom migration, was not particularly intense (see Figure 4), requiring long acquisition times to detect. For C^+^, the recording time was 5500 s, while, for H_3_^+^ cations, it was 600 s. Moreover, identifying luminescence originating from an OH fragment requires precise computer simulations, as demonstrated in the study [13]. Nevertheless, as seen in Figure 4, the 305–308 nm range shows a clear part of the OH(A^2^Σ^+^→X^2^Π) band. Simulations indicate that the R branch head of the (0,0) transitions generates peaks in this region. Other Δν = 0 transitions of OH(A^2^Σ^+^→X^2^Π) overlap with the CH(C^2^Σ^+^→X^2^Π_r_) band.

We previously observed ion-induced hydrogen migration in collisions of oxygen cations with tetrahydrofuran molecules [68]. In this case, an undisturbed OH(A^2^Σ^+^→X^2^Π) Δν = 1 emission spectrum was clearly visible within the 280.6–297.6 nm wavelength range. Furthermore, we observed this process in collisions of pyridine with the H^+^, H_2_^+^, He^+^, He^2+^ wavelength range [21]. In this example, we demonstrated that the production of NH(A^3^Π) fragments depended on both the type and velocity of the ion, allowing modulation of the hydrogen transfer process. The hydrogen migration was also observed in the photodissociation of pyridine [67], tetrahydrofuran [69], and isoxazole [70]. Moreover, Upadhyaya’s recent calculations showed [71] that thiophene (C_4_H_4_S, having S heteroatom in the ring instead of the oxygen) can undergo at least three distinct types of hydrogen atom migration, which may be classified as primary processes.

Although theoretical models predict hydrogen migration in furan between carbon atoms, potentially leading to CH or CH_2_ fragment formation, our optical spectroscopy data do not reveal emission signatures of this mechanism. In particular, no features were observed in the 550–600 nm region corresponding to the well-characterized CH_2_ radical emission in the visible region of ^1^B₁ → ^1^A₁ transition observed in the 550.06–751.88 nm region [72]. Thus, while such pathways are theoretically plausible, they remain spectroscopically unconfirmed under our experimental conditions.

It is of note that investigations into the fragmentation dynamics of the amino acids glycine [73] and alanine [74] under keV ion impact have revealed molecular rearrangements that resulted in the formation of NH_3_^+^ and H_3_^+^ ions, respectively, providing evidence for hydrogen migration during the fragmentation process. Furthermore, several collisional studies involving simple hydrocarbons [75] and methanol [76,77] have indicated a significant role of hydrogen migration in the fragmentation processes of these molecules. Therefore, hydrogen migration appears to be one of the key processes associated with reorganizing chemical bonds and atomic positions in many different types of molecules, thereby facilitating the redistribution of energy among the available degrees of freedom.

## 4. Materials and Methods

The experiments were conducted at the University of Gdańsk using a collision-induced emission spectrometer, as shown in detail in previous studies [10,12,16,22]. A schematic view of the apparatus can be found in [16]. Here, a description including data specifically related to furan and the projectiles used is provided below.

The spectrometer utilized in this study was constructed from four distinct components, each serving a specific function. The first unit comprised a chamber where the ion source was positioned. This source operated via a hot cathode discharge of a Colutron type, which utilized various gases under specific pressure conditions. In the present experiment, molecular hydrogen gas (H_2_, Linde, Chelmsford, MA, USA, purity 99.999%) was employed to generate trihydrogen cations (H_3_^+^) and methane (CH_4_, Linde, purity 99.5%) to produce carbon (C^+^) ions. The source was maintained at a pressure of 100 Pa for both incident gases. The ionization process occurred in a region where the distance between the cathode and anode was fixed at 5 mm, optimizing the plasma conditions for ion production. The anode-to-cathode voltage was maintained at 100 V, with a discharge current of 500 mA. By manipulating this voltage, it is possible to precisely control the ion beam characteristics, which is essential for experiments requiring specific ion reactivity and behavior. Specifically, the voltage level between the anode and cathode significantly influenced the formation of metastable cations. Lower voltages in the ion source were associated with a reduction in metastable ions in the cation beam. However, to minimize the amount of H_3_^+^ and C^+^ metastables and to still have efficient discharge, we maintained a relatively very high ion source pressure of 100 Pa, as mentioned above.

The ions generated in the plasma were extracted by applying a 1000 V potential and then directed toward the second component of the experimental setup. This unit was equipped with a 60° magnetic mass selector utilizing a magnetic field to deflect ions. The degree of deflection was proportional to their *m*/*q* ratio, thus enabling the selection of cations that, in the next step, underwent energy adjustments through an electrostatic lens system, optimizing their kinetic energy for the subsequent collisional process. The beam currents at 1000 eV were about 30 nA for carbon cations. The cation beam current of H_3_^+^, measured at the rear slit of the collision cell, exhibited a strong dependence on the ion energy. Typically, the current decreased significantly—by a factor of approximately 125—when the energy of H_3_^+^ was reduced from 1000 eV to a range of 50 eV. Specifically, at an energy of 50 eV, the cation beam current in the collision region was approximately 1.6 nA for H_3_^+^, whereas, at 1000 eV, the current increased to 200 nA. Therefore, the cation beam current was simultaneously recorded for normalization purposes.

In the collision cell, the cation beams interacted with furan vapors, resulting in the fragmentation of the furan molecules into both ionized and neutral fragments. Additionally, some fragments may originate from the excitation and fragmentation of neutralized projectiles. The luminescence emitted from these fragments was captured using an optical spectrometer located in the final part of the spectrometer. The emission lifetimes of the observed atomic and molecular products were sufficiently short to ensure that all luminescence was emitted before leaving the collision cell. The optical system had a 1024-channel “Mepsicron” multi-channel photon detector and a McPherson 218 spectrograph with interchangeable gratings, providing high spectral resolution. The detector was sensitive within the 180–600 nm wavelength range. Experimental data were collected using a 1200 lines/mm grating blazed at 250 nm or a 300 lines/mm grating blazed at 500 nm. The 1200 lines/mm grating allowed for recording high-resolution spectra (Δ*λ* = 0.4 nm, full width at half maximum, FWHM), which was suitable for identifying all emitting features. However, the scans measured with this grating covered only a 40 nm wavelength window. Moreover, the acquisition times were considerably long for this grating due to the low emission intensities. Hence, high-resolution spectra were only obtained for projectile energies of 1000 eV. The 300 L/mm grating was used to collect more light over nearly 200 nm wavelength windows and measure emission yields, representing the relative emission cross-sections (*σ*) corresponding to the formation probabilities of the observed products at a given energy. The dispersed spectra were characterized by lower optical resolution (Δ*λ* = 2.5 nm), which was still sufficient to distinguish individual luminescence features. In order to determine the cross-sections for the production of a given product, the optical spectrometer was set to a selected spectral range covering the chosen atomic line or molecular band to record luminescence at a precisely defined wavelength as a function of the incident cation energy. The luminescence measurement at each energy was repeated numerous times to ensure reliable statistics. The background signal was also measured by blocking the furan beam and then subtracted from each optical fragmentation spectrum to ensure residual gas contaminations are subtracted from the original data. Next, each spectrum was corrected for the wavelength dependence of the detection system’s sensitivity, which had previously been determined for the 300 L/mm grating using standard monochromatized light sources [78]. Finally, the cross-sections of the specific product were obtained by integrating over the peak or band area and normalizing to recording time, cation beam current, and pressure. Notably, the production of carbon cations quickly deteriorated the cathode when attempting to achieve high beam currents. Therefore, it was decided not to carry out cross-section measurements with C^+^ ions, as these could be time-consuming due to the relatively low C^+^ beam currents maintained in this study.

Furan, purchased from Sigma-Aldrich with a declared purity of 99%, is a volatile liquid (with a vapor pressure of 493 mmHg at 20 °C [79]) and was used without heating. It was degassed using multiple freeze–pump–thaw cycles. Efforts were made to ensure linear dependences between the emission signal and the furan gas pressure to avoid collisional quenching of excited fragments, trapping of the emitted radiation, or double cation collisions. Specifically, it was confirmed that the emission signal increased linearly with pressure up to 30 mTorr, confirming that the system was operating within a single-collision regime. Therefore, the pressure of furan in the system was maintained at the midpoint of the studied range, i.e., at a constant 15 mTorr, monitored by a Barocel capacitance manometer.

Notably, the atomic lines observed in the spectra were identified using data from the NIST database [43]. In contrast, the identification of molecular bands required theoretical simulations. Therefore, theoretical spectra for the CH(A^2^Δ→X^2^Π_r_; B^2^Σ^+^→X^2^Π_r_; C^2^Σ^+^→X^2^Π_r_) Δ*ν* = 0 and OH(A^2^Σ^+^→X^2^Π) molecular bands were calculated using the LIFBASE molecular spectra simulation package [45]. Spectral fittings were then performed by comparing experimental data with the simulations generated. The simulations for the CH and OH spectra utilized the relevant spectroscopic vibrational and rotational constants of the A^2^Δ, B^2^Σ^+^, C^2^Σ^+^, and X^2^Π_r_ electronic states of CH [80,81,82,83,84], as well as the A^2^Σ^+^ and X^2^Π states of OH [85,86,87,88]. The line positions and intensities were calculated using these constants, and the vibrational and rotational populations were assumed to follow a Boltzmann distribution governed by the characteristic vibrational (*Tᵥ*) and rotational (*T_R_*) temperatures. Additionally, the Voigt profile for the apparatus function, with a resolution of Δ*λ* = 0.4 nm (FWHM), was applied in the simulations. The temperature errors represent the maximum fitting uncertainties observed when *Tᵥ* and *T_R_* were varied, resulting in a 0.5% deviation from the optimal peak correlation and χ^2^ values.

## 5. Conclusions

The experimental setup we utilized enabled a detailed investigation of the fragmentation and emission processes occurring during the interaction of furan molecules with energetic H_3_^+^ and C^+^ cations, providing insights into the mechanisms underlying ion-induced dissociation in heterocyclic oxygen-containing molecules. Specifically, a novel gas-phase study was performed involving collisions of furan (C_4_H_4_O) with H_3_^+^ and C^+^ ions at laboratory-frame impact energies ranging from 50 to 1000 eV. Collision-induced emission spectroscopy carried out at 1000 eV revealed high-resolution fragmentation spectra for both ion species. While the emission patterns were broadly similar, collisions with C^+^ led to significantly more extensive molecular fragmentation. Relative emission cross-sections for selected fragments were quantified in the H_3_^+^–furan system. The present results were analyzed in relation to prior collisional studies on furan and related heterocyclic molecules. The data highlight the influence of both projectile type and target structure on fragmentation dynamics and energy redistribution. For furan, THF, and pyridine, analysis of hydrogen and CH radical emission revealed that increasing projectile complexity (H^+^ < H_2_^+^ < H_3_^+^) leads to decreased H and increased CH emission, indicating enhanced fragmentation. THF showed higher H and lower CH emission compared to furan and pyridine, likely due to its saturated structure, weaker C–H bonds, and high cross-section for charge transfer (CT) reactions. The He^+^ collisions with furan and pyridine resulted in stronger CH emission and more structured fragmentation spectra, likely due to higher energy transfer to the target in the absence of electron capture. C^+^ + THF interactions led to dominant carbon emission, suggesting direct C atom knockout from the furan skeleton. Moreover, He^2+^ ions caused less fragmentation than He^+^ at the same kinetic energy, inducing softer collisions that resulted in fewer and less intense fragment ion signals compared to collisions with He^+^ cations. These observations agree with general trends identified by mass spectrometry in ion collisions with furan and other complex molecules, as well as with those predicted by quantum chemical calculations.

Investigating the fragmentation of furan under ionic impact provides insights into radiation-induced molecular degradation, with implications for astrobiology, radiobiology, and medicine. Understanding bond breakage during ion collisions not only aids in validating theoretical models of DNA damage but also improves the design and stability assessment of furan-containing drugs and molecular probes in ionizing environments. Moreover, furan derivatives such as furfural and 5-hydroxymethylfurfural (HMF) are key intermediates in biomass-to-fuel conversion. Ion–furan interaction studies can, therefore, give insight into reaction pathways relevant to optimizing green chemistry and sustainable fuel production. Additionally, the behavior of furan under ion impact is relevant for modeling conditions in combustion, plasma processing, and planetary atmospheres, particularly in relation to pollutant formation and reaction kinetics. In space environments, ions like H_3_^+^ and C^+^ are abundant. C^+^ and H_3_^+^ ions at 50–1000 eV correspond well to the velocity range of stellar winds from young or low-to-intermediate mass stars, shocks, or disk winds [89,90]. Thus, experiments involving furan and low-energy ions can simulate interactions between stellar winds and organic molecules in interstellar clouds, comets, and planetary atmospheres, which is essential for astrochemical models and the search for prebiotic chemistry. Furthermore, given their established utility in estimating interstellar dust extinction in star-forming galaxies [91] or diagnosing high-power plasmas [92], Balmer line intensity ratios (Balmer decrements) represent a valuable diagnostic method for probing the local plasma conditions and potential dust-related attenuation effects, providing insight into both intrinsic emission mechanisms and interactions with surrounding environments (e.g., dust or surfaces), enhancing our understanding of physical processes in laboratory and astrophysical plasmas.

## Figures and Tables

**Table 1:** The values of vibrational (*T*_v_) and rotational (*T*_R_) temperatures obtained from the simulations. For CH(A^2^Δ) and CH(B^2^Σ^+^), the *T_v_* and *T_R_* uncertainties are estimated to be 500 and 200 K, respectively, while for CH(C^2^Σ^+^) and OH(A^2^Σ^+^), these uncertainties were 500 and 500 K.

Transition	H_3_^+^ + C_4_H_4_O		C^+^ + C_4_H_4_O	
	*T*_v_ [K]	*T*_R_ [K]	*T*_v_ [K]	*T*_R_ [K]
CH(A^2^Δ→X^2^Π_r_) Δν = 0	7700	4150	8000	5000
CH(B^2^Σ^+^→X^2^Π_r_) Δν = 0	3000	3500	3000	5000
CH(C^2^Σ^+^→X^2^Π_r_) Δν = 0	4900 *	5500 *	5500 *	6000 *
OH(A^2^Σ^+^→X^2^Π) Δν = 0	3900 *	4800 *	10,000 *	12,000 *

* Data from Reference [13].

**Table 2:** The emissions observed in the C^+^ and H_3_^+^ collisions with furan. The positions (λ) of the band heads or the centers of the atomic lines are also given.

Product	Electronic Transition	Vibrational Transition	λ (nm)	References
CH	A^2^Δ→X^2^Π_r_	(0;0)	431.0	[45,46]
		(1;1)	431.0	
		(2;2)	432.5	
		(3;3)	435.7	
	B^2^Σ^+^→X^2^Π_r_	(0;0)	386.5	
	C^2^Σ^+^→X^2^Π_r_	(0;0)	314.5	
		(1;1)	316.0	
OH	A^2^Σ^+^→X^2^Π	(0;0)	306.5	[45,46]
		(1;1)	312.2	
H	*n* = 4→2	-	486.1	[47]
	*n* = 5→2	-	434.0	
	*n* = 6→2	-	410.2	
	*n* = 7→2	-	397.0	
	*n* = 8→2	-	386.0	
	*n* = 9→2	-	379.0	
	*n* = 10→2	-	373.0	
C	2p3s ^1^P_1_→2p^2 1^D_2_	-	193.1 (386.2 II^nd^ order)	[47]
	2p3s ^1^P_1_→2p^2 1^S_0_	-	247.9 (495.8 II^nd^ order)	

**Table 3:** Average relative abundances of emitting fragments, *RA* (in %). The results of collisions of furan [10,12,15], tetrahydrofuran [16,18,19], and pyridine [22] with different cations are shown for comparison.

	Furan (C_4_H_4_O)					Tetrahydrofuran (C_4_H_8_O)					Pyridine (C_5_H_5_N)		
	H^+^	H_2_^+^	H_3_^+^	He^++^	He^+^	H^+^	H_2_^+^	H_3_^+^	C^+^	O^+^	H^+^	H_2_^+^	He^+^
H(*n* = 4–7)	58.3	46.3	37.6	47.9	8.4	88.8	76.2	67.3	4.8	17.6	61.4	45.9	10.8
CH(A,B,C)	39.9	51.0	59.4	52.1	82.1	11.2	23.8	32.7	36.2	69.6	26.9	40.4	42.2
Other	1.9	2.7	3.0	-	9.5	-	-	-	59.1	12.8	11.7	13.7	47.0

## Data Availability

The original contributions presented in this study are included in the article. Further inquiries can be directed to the corresponding author.

## Abbreviations

The following abbreviations are used in this manuscript:

CIES	collision-induced emission spectroscopy
FWHM	full width at half maximum
CT	charge transfer
THF	tetrahydrofuran
